# Establishment of the deuterium oxide dilution method as a new possibility for determining the transendothelial water permeability

**DOI:** 10.1007/s00424-024-02934-z

**Published:** 2024-03-05

**Authors:** Hannes Müller, Janina Hahn, Angelina Gierke, Robert Stark, Cornelia Brunner, Thomas K. Hoffmann, Jens Greve, Oliver Wittekindt, Robin Lochbaum

**Affiliations:** 1 Department of Otorhinolaryngology, Head and Neck Surgery, Ulm University Medical Center, Frauensteige 12, 89075 Ulm, Germany; 2 Department of General Physiology, Ulm University, Albert-Einstein-Allee 11, 89081, Ulm, Germany

**Keywords:** Edema, D_2_O dilution method, Transendothelial water permeability, Endothelial barrier function

## Abstract

Increase in transendothelial water permeability is an essential etiological factor in a variety of diseases like edema and shock. Despite the high clinical relevance, there has been no precise method to detect transendothelial water flow until now. The deuterium oxide (D_2_O) dilution method, already established for measuring transepithelial water transport, was used to precisely determine the transendothelial water permeability. It detected appropriate transendothelial water flow induced by different hydrostatic forces. This was shown in four different endothelial cell types. The general experimental setup was verified by gravimetry and absorbance spectroscopy. Determination of transendothelial electrical resistance (TEER) and immunocytochemical staining for proteins of the cell-cell contacts were performed to ensure that no damage to the endothelium occurred because of the measurements. Furthermore, endothelial barrier function was modulated. Measurement of transendothelial water flux was verified by measuring the TEER, the apparent permeability coefficient and the electrical capacity. The barrier-promoting substances cyclic adenosine monophosphate and iloprost reduced TEER and electrical capacity and increased permeability. This was accompanied by a reduced transendothelial water flux. In contrast, the barrier-damaging substances thrombin, histamine and bradykinin reduced TEER and electrical capacity, but increased permeability. Here, an increased water flow was shown. This newly established in vitro method for direct measurement of transendothelial water permeability was verified as a highly precise technique in various assays. The use of patient-specific endothelial cells enables individualized precision medicine in the context of basic edema research, for example regarding the development of barrier-protective pharmaceuticals.

**Supplementary information:**

The online version contains supplementary material available at 10.1007/s00424-024-02934-z.

## Introduction

The endothelial barrier separates the intravascular space from the surrounding interstitium. It prevents uncontrolled exchanges of liquid, dissolved substances, and cells between these compartments. The compromise of the endothelial barrier function is implicated in the pathophysiology of various diseases, including polytrauma, sepsis, autoimmune conditions, and diabetic vasculopathy [7]. Diseases affecting endothelial barrier function also include different forms of angioedema, arising from factors such as allergies, genetic defects, or as a side effect of angiotensin-converting enzyme (ACE) inhibitors [27]. Edema manifests when there is an imbalance between increased net filtration in the capillaries, surpassing reabsorption, and lymphatic removal. This imbalance may result from heightened hydrostatic pressure in the terminal flow path or reduced intraluminal osmotic pressure [25]. Additionally, the breakdown of the endothelial barrier constitutes a significant contributory factor to edema [23]. Under physiological conditions, the endothelial barrier is maintained through the formation of cell-cell contacts among endothelial cells. Of particular relevance are the adherens junctions (AJ) and the tight junctions (TJ) [19, 37], as well as the glycocalyx [11].

During increased permeability there is a reorganization of cortically aligned actin bundles, transforming them into radially aligned filaments. This process gives rise to the formation of focal adhesion contacts derived from the continuous adherens junctions. The selective distribution of AJ, thus achieved, contributes significantly to the overall increase in permeability [31]. Furthermore, alterations in the expression profile of specific tight junction (TJ) proteins have been observed [23].

To assess endothelial barrier function, various methods can be employed, including the measurement of transendothelial electrical resistance (TEER) [5], the apparent permeability coefficient (P_app_) [38] or the electrical capacity [17]. Existing methods for directly measuring transendothelial water flow include gravimetry and absorption spectroscopy. Gravimetry quantifies the mass of the apical liquid and utilizes fluid density to calculate volume. However, both methods are susceptible to errors. For instance, the formation of liquid meniscus results in varying heights of the water column, and precise pipetting without damaging the cell layer poses technical challenges [33, 36]. Other approaches involve fluorometric quantification of marker substances, where changes in concentration due to volume shifts in the liquids provide insights into alterations in volumes. However, there is a risk of cellular metabolism affecting marker substances or uneven distribution in the liquid [10, 22, 26]. These challenges underscore the current lack of an exact and error-proof method for determining transendothelial water flow, despite its clinical significance.

A method devoid of the limitations previously mentioned is the D_2_O dilution method. Initially designed for investigating transepithelial fluid transport, this method capitalizes on the distinct absorption properties of water and deuterium oxide, the latter possessing an additional neutron in the nucleus. Notably, this method has demonstrated reduced susceptibility to errors when examining transepithelial water transports and has showcased nanoliter resolution [33]. Consequently, it is anticipated that the D_2_O dilution method will also prove suitable for investigating transendothelial water fluxes.

## Methods

### Cell culture

The experiments were performed with different cell types to investigate endothelial cell function. Human umbilical vein endothelial cells (HUVEC), human umbilical artery endothelial cells (HUAEC) and human dermal microvascular endothelial cells (HDMEC) were all obtained from *Promocell GmbH, Heidelberg*. The cells were cultivated in endothelial cell growth medium with fetal calf serum (FCS, 0.02 ml/ml), epidermal growth factor (EGF, 5 ng/ml), basic fibroblast growth factor (FGF, 10 ng/ml), insulin-like growth factor (IGF, 20 ng/ml), vascular endothelial growth factor (VEGF, 0.5 ng/ml), ascorbic acid (1 µg/ml), hydrocortisone (0.2 µg/ml), heparin (22.5 µg/ml) and 2% penicillin/streptomycin (all from *Promocell GmbH, Heidelberg*). Additionally, blood outgrowth endothelial cells (BOEC) were isolated and cultivated from peripheral venous blood of healthy probands according to the original protocol [29]. Every experiment was performed with at least two different cell batches or donors; cells were used till passage 3. The cells were seeded on semipermeable transwell filter inserts with a pore size of 0.4 µM *(Sarstedt AG & Co. KG, Nümbrecht*) in 24 well cell culture plates, which had been previously coated with collagen solution (*Thermo Fisher Scientific, Karlsruhe*). Medium was changed at day two after cultivation on filters. Unless specified otherwise, all experiments were conducted on the third day. The following modulators were used at the indicated concentration: 8-bromo-adenosine-3',5'-cyclophosphate (0.25 mg/ml), histamine (0.1 nMol/ml), bradykinin (0.1 µMol/ml), thrombin (1 U/ml) all from *Sigma-Aldrich Chemie GmbH, Steinheim*, icatibant (10 nM) was obtained from *Takeda Pharma GmbH, Berlin*.

### D_2_O dilution method

On day three after seeding the cells on transwell filters, filters were placed in a fresh 24 well plate. Previously, confluency of the monolayer was assessed using phase contrast microscopy and measurement of transendothelial electrical resistance (TEER). Isotonic saline (*Fresenius Kabi, Bad Homburg*) was placed in the apical compartment and the corresponding culture medium in the basal compartment. To induce water flow, specific volumes were added to the respective compartments, as detailed in Table 1.

**Table 1:** Volumes used to generate the hydrostatic gradients

Hydrostatic pressure difference from apical to basal				
Water column [cm]	0	0.5	0.75	1
Volume apical [µl]	100	363	477	636
Volume basal [µl]	710	454	454	454
Hydrostatic pressure difference from basal to apical				
Water column [cm]	0	0.5	0.75	
Volume apical [µl]	100	100	100	
Volume basal [µl]	710	1525	1932,5	

Modulators were added both apically and basally. After the respective incubation time, the volume of liquid to be analyzed was mixed well apically with 25 µL of isotonic D_2_O solution (*Sigma-Aldrich Chemie GmbH, Steinheim*) and subsequently transferred to a reaction tube, securely sealed to prevent proton exchange with ambient humidity. The volume of the resulting mixture of H_2_O and D_2_O was then analyzed by attenuated total reflection midinfrared spectroscopy. A Fourier transform infrared spectrometer with an ALPHA II Fourier-transform infrared spectrometer with an Eco-ATR unit (both from *Bruker optics, Ettlingen*) was used for further analysis. For each series of measurements, a calibration series was prepared using 5%, 15%, 25%, 40%, 50%, 65%, 75%, 85%, and 95% H2O in D_2_O. Then, 15 µl of the sample was measured with 100 scans in a wavenumber of 4000 to 400 cm^−1^ with a spectral resolution of 4 cm^−1^. By integration, the area under the absorption bands (H–O stretching vibration band: 3810.6 cm^−1^—2805 cm^−1^; D-O stretching vibration band: 2774 cm^−1^—2070 cm^−1^; D-O-D bending vibration band: 1818 cm^−1^—1090 cm^−1^) was determined and plotted against the known water concentration of the calibration series. For each of the above absorption bands, the fraction of H_2_O was calculated using the following formula: *rel*_*H2O*_ = *m x c / Int*. Where *rel*_*H2O*_ = percentage of H_2_O, *m* = gradient of the regression line, *c* = y-axis intercept of the regression line and *Int* = integral of the absorption band. With the water fraction thus determined, the apical volume was calculated: *V*_*H2O*_ = *rel*_*H2O*_* x V*_*D2O*_* / (100 – rel*_*H2O*_*)*. Where *V*_*D2O*_ = volume D_2_O (= 25 μl).

### Immunocytochemistry

Following cell cultivation on transwell filters, cells underwent two washes with phosphate-buffered saline containing calcium and magnesium (PBS, *PAN-Biotech GmbH, Aidenbach*) and were subsequently fixed using 4% formaldehyde (*Thermo Fisher Scientific, Karlsruhe*) in PBS for a duration of 10 to 20 min. After three additional washes with pure PBS, cells were treated with 0.1% Triton X-100 (*Sigma Aldrich Chemie GmbH, Steinheim*) in PBS for 5 min, followed by two 5-min incubations in 0.5% Triton X-100 with 2% fetal calf serum (FCS) in PBS. Primary antibodies targeting vascular endothelial cadherin (VE-cadherin Polyclonal Antibody, Rabbit IgG, 36–1900, *Thermo Fisher Scientific, Karlsruhe*) and zonula occludens protein 1 (Anti- ZO1 tight junction protein antibody, Rabbit, ab221547, *Abcam, Cambridge*) were diluted 1: 100 in PBS with 2% FCS and applied to the cells for 1 h at room temperature. After three washes with PBS containing 2% FCS, a secondary antibody (Donkey anti-Rabbit IgG (H + L), secondary antibody, Alexa Fluor 488, A-21206, *Thermo Fisher Scientific, Karlsruhe*) was diluted 1: 400 in PBS with 2% FCS. For nuclear staining Hoechst 33342 (*Thermo Fisher Scientific, Karlsruhe*) was added in a 1: 50,000 dilution. Cells were incubated in this solution for 1 h at room temperature. After 3 washing steps using PBS with 2% FCS and 3 with pure PBS, filters were transferred on ibiTreat μ-Slide 8 Well chambers (*Ibidi GmbH, Martinsried*). Microscopy was performed with the Fluorescence Microscope KEYENCE BZ-9000 (*Keyence Deutschland GmbH, Neu-Isenburg*), image analysis with FIJI based on ImageJ (*National Institutes of Health, Bethesda*).

### Measurement of the transendothelial electrical resistance

Transwell filters were transferred to the cellZScope (*nanoAnalytics GmbH, Münster*) on day three after seeding and 250 µl of appropriate medium was added to the apical compartment and 500 µl to the basal compartment. Analysis was performed using the according software.

### Measurement of the apparent permeability coefficient (P_app_)

P_app_ was determined for fluorescein isothiocyanate-coupled dextrans with a size of 70 kDa (*Sigma-Aldrich Chemie GmbH, Steinheim*). On day three, 100 µl isotonic saline were added apically and 500 µl culture medium basally. The dextranes were also added basally. After one hour the medium was removed from the apical compartment. A dilution series with ascending known concentration of the dextrans was also prepared. The intensity of the fluorescence was determined with the Infinite M200 plate reader measuring instrument (*Tecan Group AG, Männedorf*) and the corresponding concentration of the dextrans was calculated using the calibration line. Subsequently, the P_app_ was calculated using the following equation: *P*_*app*_ = (*C* × *V*_0_)/(∆*t* × *A* × ∆*C*_0_). With *C* = concentration of marker substance after incubation time, *V*_*0*_ = apical volume of isotonic saline at baseline, *∆t* = incubation time, *A* = area of the filter, *∆C*_*0*_ = difference in concentration of marker substance between apical and basal compartment at baseline (t = 0).

### Real time cell analysis (RTCA)

The xCelligence-RTCA (*Agilent Technologies Germany GmbH & Co. KG, Waldbronn*) and associated E-plates were used for these measurements. The plates are equipped with gold electrodes. Cells are electrical insulators and so impede the flow of charged particles. This is measured as the electrical capacitance and is indicated by the cell index. Cells were transferred to electrodes and incubated for three days. When necessary, barrier-modulating substances were then added and changes in the so-called cell index were read as a measure of the electrical capacity using the corresponding software.

### Gravimetry

On the third day after cultivation, the medium in the apical and basal compartments was aspirated. An appropriate hydrostatic force (see D_2_O dilution method) was then applied. Subsequently, the entire apical volume intended for examination was carefully transferred by pipetting, and the pipetted fluid was weighed using a precision balance (*Sartorius AG, Göttingen, Germany*). The sought volume was then calculated as follows: *MVF* = *M*_total_—*MRG* and *VF* = *MFV*/ρ_*H*2*O*_. Where MFV = mass of liquid volume [g], M_total_ = total mass [g], MRG = mass of empty reaction vessel [g], VF = liquid volume [ml] and ρ_H2O_ = density of water [g/ml] (= 1 g/ml).

### Absorption spectroscopy

Phenol red-free culture medium (*Promocell GmbH, Heidelberg, Germany*) was used for absorption spectroscopy. On day 4, the culture medium was removed from both compartments, and these were washed with PBS (*PAN-Biotech, Aidenbach, Germany*) and the corresponding hydrostatic gradients were applied. In addition, phenol red (30 mg/L, Sigma-Aldrich Chemie GmbH, Steinheim, Germany) was added to one of the two compartments. Phenol red, being unable to permeate the endothelial cell layer, remained confined to the compartment in which it was added. The intensity of the absorption of phenol red was measured at a wavelength of a wavelength of 479 nm by absorption spectroscopy using the Infinite M200 plate reader instrument. The water flux was finally calculated as given in [22].

### Statistical analysis

Prism 5 (*GraphPad, San Diego*) was used for data analysis. Statistical tests used are given in the text. Differences were considered statistically significant when *p* < 0.05, and significance levels were depicted in the figures as follows: *p < 0.05, **p < 0.01, ***p < 0.001.

## Results

### Development of an experimental setup for the determination of transendothelial water movement *in vitro*

**Fig. 1:**
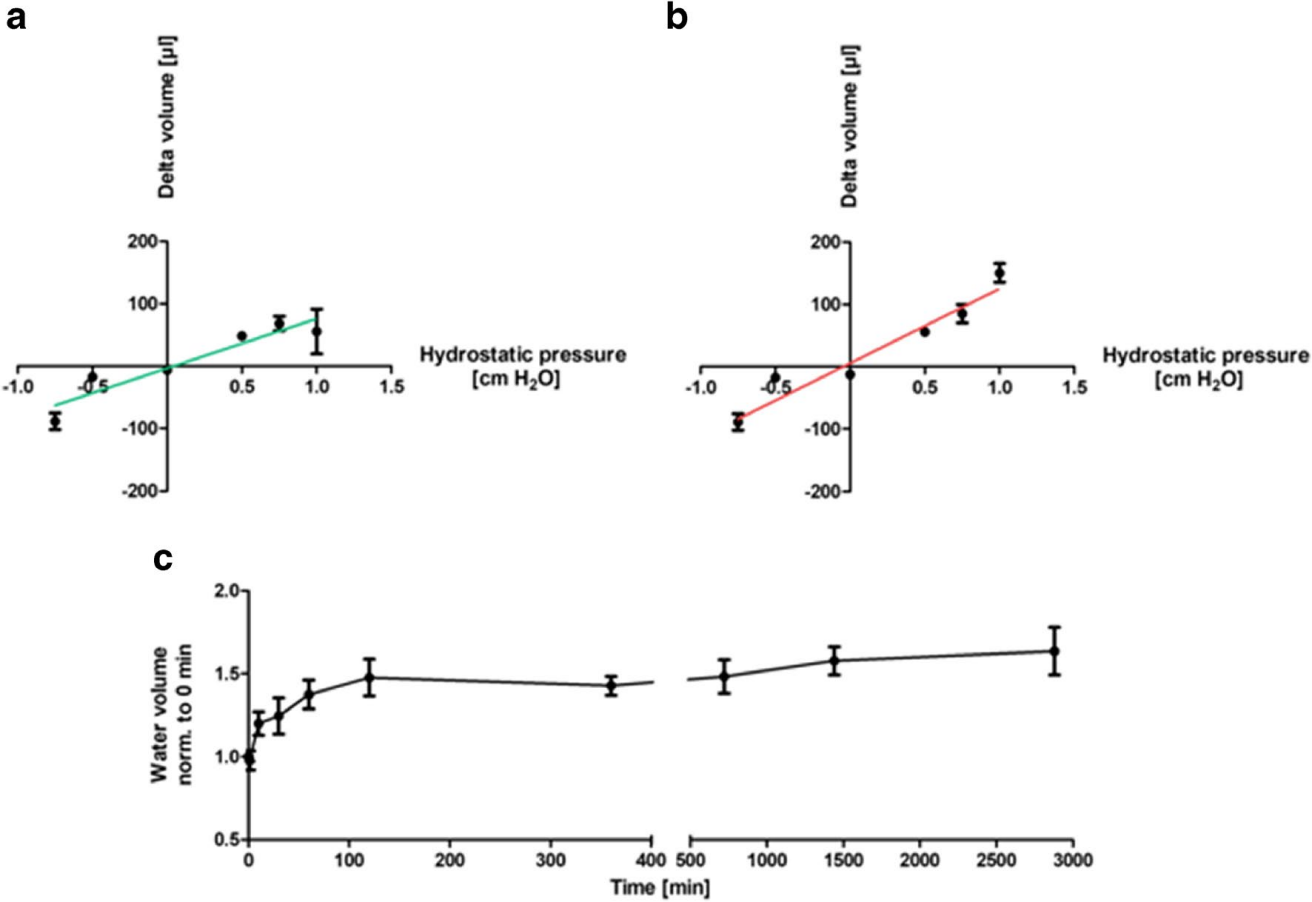
Dependence of transendothelial water movement on hydrostatic pressure gradients. Human umbilical vein endothelial cells were cultured on semipermeable transwell filter systems and exposed to different hydrostatic pressures on day three. A positive pressure is thereby directed from apical to basal, a negative one from basal to apical. Volume of the transendothelial moved fluid was determined by D_2_O dilution method; a positive value corresponds to a flow toward basal, a negative toward apical. **a**/**b** show the dependence of water movement on different hydrostatic pressures after 6 h (**a**) and 24 h (**b**) of incubation. The regression line shows a proportional relationship between the pressure gradient and the transendothelial water flow. Using Spearman's two-tailed correlation analysis, a significant correlation between pressure and water flow was shown for both study conditions (**a**: r = 0.9429, *p* = 0.0167; **b**: r = 1.0000, *p* = 0.0028). Time course experiments show the time dependency of transendothelial water movements (**c**). Values are given as mean ± standard deviation

HUVEC are a widely accepted endothelial cell type. The cells were therefore used as an exemplary cell model to establish the experimental setup for determination of transendothelial water permeability. These were cultured on semipermeable transwell filters as described in the methods. Initially, different hydrostatic pressures were applied basally and apically to determine the optimal measurement conditions. Measurements were performed after 6 and 24 h. A water flow from apical to basal was determined as positive, from basal to apical as negative (see Fig. 1). A positive hydrostatic pressure represented a gradient directed from apical to basal, a negative pressure one directed from basal to apical. Hydrostatic pressures showed a linear relationship between applied pressure and water flow. Fluid flows from apical to basal as well as in the other direction according to the hydrostatic pressure gradient could be detected. Optimal pressure/time ratios were set at 1.0 cmH_2_O / -0.75 cmH_2_O and 24 h. Afterwards, time course measurements with 1.0 cmH_2_O were performed (c).

### Hydrostatic pressure does not damage endothelial monolayer integrity

**Fig. 2:**
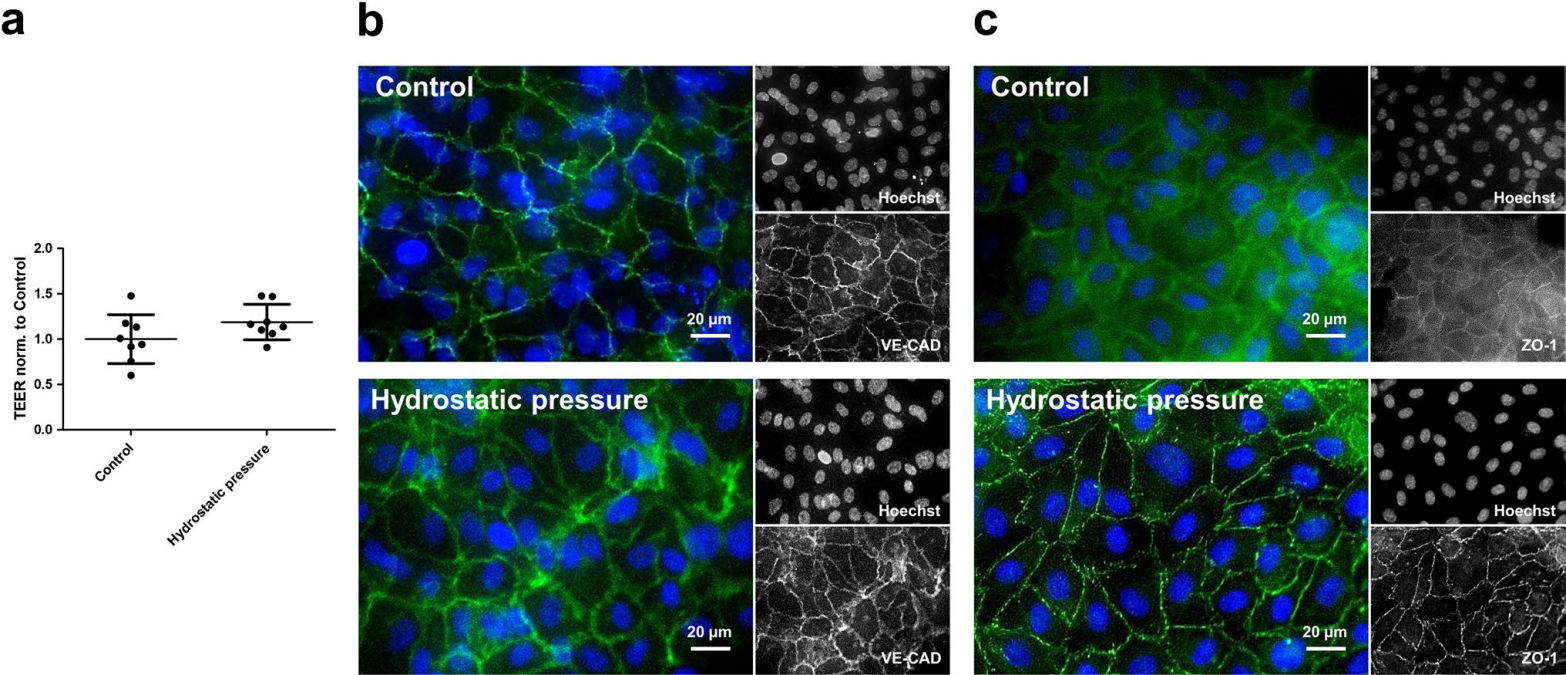
Evaluation of endothelial monolayer integrity after application of hydrostatic pressure. Human umbilical vein endothelial cells were cultured on semipermeable transwell filter systems and exposed to a hydrostatic pressure of 1.0 cmH2O apically on day three. To investigate if hydrostatic pressure disturbs endothelial cell integrity, transendothelial electrical resistance (TEER) was measured before and after application of the pressure gradient (**a**). Here, no significant changes were detected. Also, immunocytochemical staining of cell-cell contact proteins vascular endothelial cadherin (VE-CAD, **b**) and zonula occludens protein 1 (ZO-1) was performed. The colored images show the cell-cell contact protein in green and nuclear staining with Hoechst in blue. Values are shown as scattered plots, and the mean value is additionally indicated with the standard deviation. Each data point represents one filter. Significances were calculated using the two-tailed Mann Whitney test (* = *p* < 0.05, ** = *p* < 0.01, *** = *p* < 0.001)

To confirm that applied hydrostatic pressure does not disturb integrity of endothelial monolayer, TEER measurements and immunocytochemistry were performed before and after application of the gradient. TEER did not change at all (Fig. 2a). Staining of cell-cell contact proteins vascular endothelial cadherin (VE-CAD, Fig. 2b) and zonula occludens protein 1 (ZO-1, Fig. 2c) showed that the endothelial monolayer was still intact after application of hydrostatic pressures.

### D_2_O dilution method detects transendothelial water movement in different endothelial cell types

**Fig. 3:**
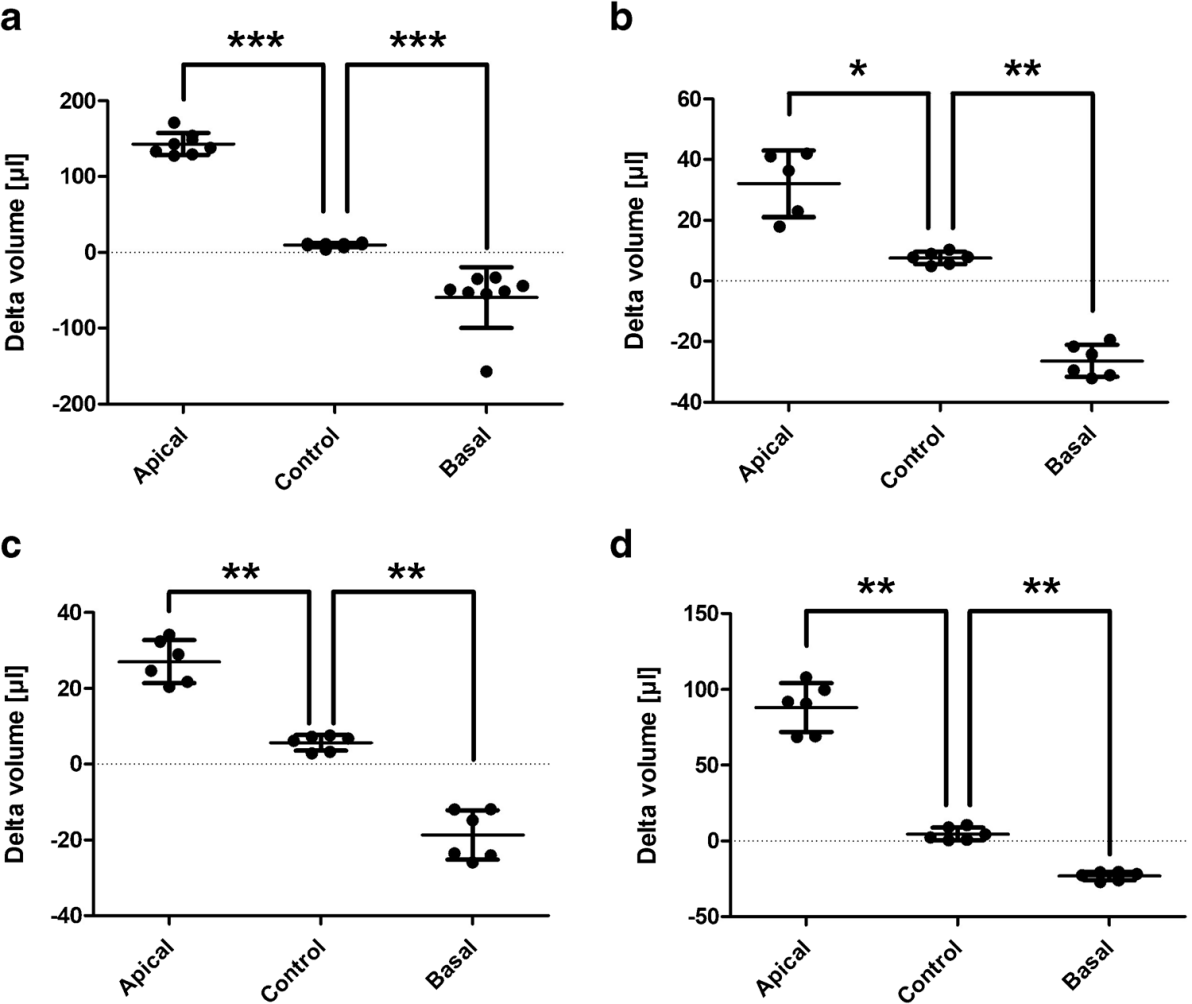
The D_2_O dilution method is able to detect transendothelial water movements from distinct endothelial cell types. Different endothelial cell cultures were cultured on semipermeable transwell filter systems and exposed to a hydrostatic pressure of 1.0 cmH_2_O apically and 0.75 cmH_2_O basally on day three. A control group without applied hydrostatic pressure was also examined. Volume of the transendothelial moved fluid was determined by D_2_O dilution method; a positive value corresponds to a flux toward basal, a negative toward apical. **a** shows the results of human umbilical vein endothelial cells, **b** of human umbilical artery endothelial cells, **c** of human dermal microvascular endothelial cells and **d** of blood outgrowth endothelial cells. All four endothelial cell cultures showed measurable water movement in the corresponding direction for each pressure gradient. Values are shown as scattered plots, and the mean value is additionally indicated with the standard deviation. Each data point represents one filter. Significances were calculated using the two-tailed Mann Whitney test, in case of multiple testing a Bonferroni correction was additionally performed (* = *p* < 0.05, ** = *p* < 0.01, *** = *p* < 0.001)

Subsequently, the specified pressures were applied to different endothelial cell types and moved fluid volume was determined using the D_2_O dilution method. In addition to venous HUVEC (Fig. 3a), arterial HUAEC (Fig. 3b), lymphatic HDMEC (Fig. 3c), and BOEC isolated from healthy volunteers (Fig. 3d) were used. In all endothelia, significant water movements in the direction of the hydrostatic pressure gradient were detected.

### Other methods for determining transendothelial water movements confirm functionality of the experimental setup

**Fig. 4:**
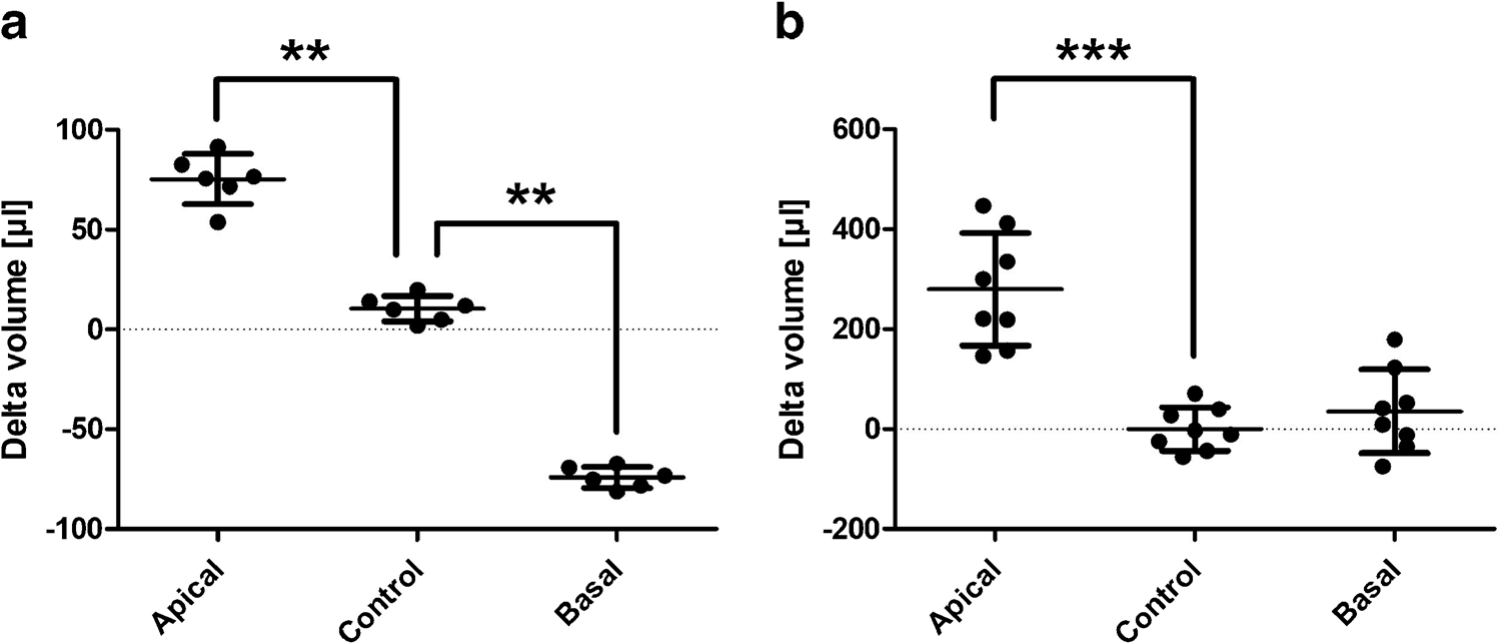
Verification of experimental setup for the D_2_O dilution method with already established methods. Human umbilical vein endothelial cells were cultured on semipermeable transwell filter systems and exposed to a hydrostatic pressure of 1.0 cmH_2_O apically and 0.75 cmH_2_O basally on day three. Volume of the transendothelial moved fluid was determined by gravimetry (**a**) and absorption spectroscopy (**b**), a positive value corresponding to flow toward the basal, a negative toward the apical compartment. Significant water fluxes could be determined for all conditions except for the basal-directed hydrostatic pressure in absorption spectroscopy. The values are shown as scattered plots, and the mean value with the standard deviation is also given. Each data point represents one filter. Significances were calculated using the two-tailed Mann Whitney test, in case of multiple testing a Bonferroni correction was additionally performed (* = *p* < 0.05, ** = *p* < 0.01, *** = *p* < 0.001)

To further verify the experimental setup itself, existing methods for determining transendothelial water movements were applied. Gravimetry (Fig. 4a) also showed a water movement corresponding to the gradient at the hydrostatic pressures. While this largely corresponded to the water flux measured in the D_2_O dilution method with a gradient from basal to apical, it was significantly lower in comparison to the reverse gradient. Absorption spectroscopy also detected water movement at apically elevated hydrostatic pressure (Fig. 4b). The delta volume was considerably higher compared to the values of D_2_O dilution and gravimetry. No water movement could be detected at basally elevated hydrostatic pressure.

### Barrier-promoting modulators decrease transendothelial water movement

**Fig. 5:**
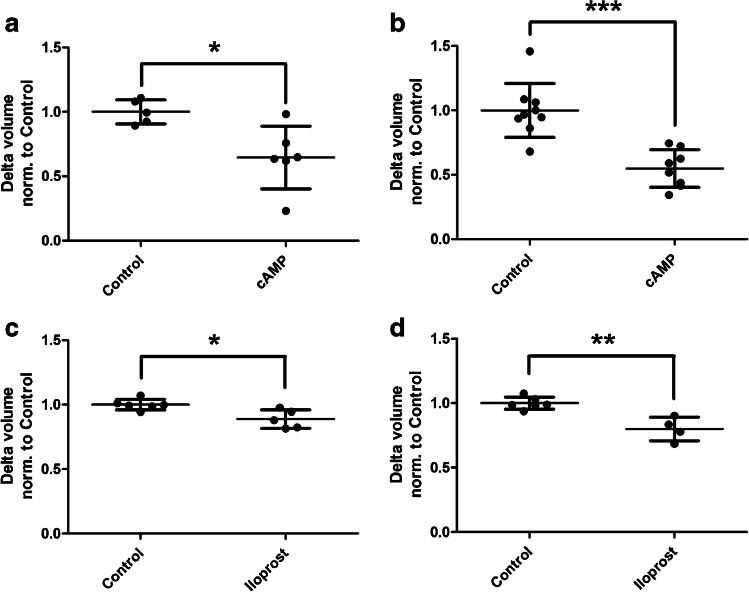
The D_2_O dilution method detects decreased water movements under barrier-protective modulators. Human umbilical vein endothelial cells were cultured on semipermeable transwell filter systems and exposed to a hydrostatic pressure of 1.0 cmH_2_O apically (**a**/**c**) and 0.75 cmH_2_O basally (**b**/**d**) on day three. Furthermore, the modulators 8-bromo-adenosine-3′,5′-cyclophosphate (cAMP, **a**/**b**) and iloprost (**c**/**d**) were added four hours before measurement. Volume of the transendothelial moved fluid was determined by D_2_O dilution method. For all conditions, a significantly decreased water movement could be detected by addition of the modulators. The values are shown as scattered plots, and the mean value with the standard deviation is also given. Each data point represents one filter. Significances were calculated using the two-tailed Mann Whitney test (* = *p* < 0.05, ** = *p* < 0.01, *** = *p* < 0.001)

The membrane-permeable cAMP analogue 8-bromo-adenosine-3′,5′-cyclophosphate (abbreviated to 8Br-cAMP), as well as the prostaglandin iloprost, are known promoters of the endothelial barrier. These two factors were thus used to detect decreased transendothelial water movement. For this purpose, hydrostatic pressure was, again, applied from apical to basal (Fig. 5a/c) and from basal to apical (Fig. 5b/d). The addition of 8Br-cAMP significantly decreased the water movement in both pressure gradients (Fig. 5a/b). The most significant reduction was seen for the hydrostatic gradient to apical, where a reduction of approximately 40% was detected. Subsequently, iloprost was used, and a significant reduction in water movement was also detected for each of the two gradients (Fig. 5c/d). Overall, however, this reduction was less pronounced than with 8Br-cAMP.

### The barrier-promoting effect of 8Br-cAMP and iloprost can be demonstrated by additional methods

**Fig. 6:**
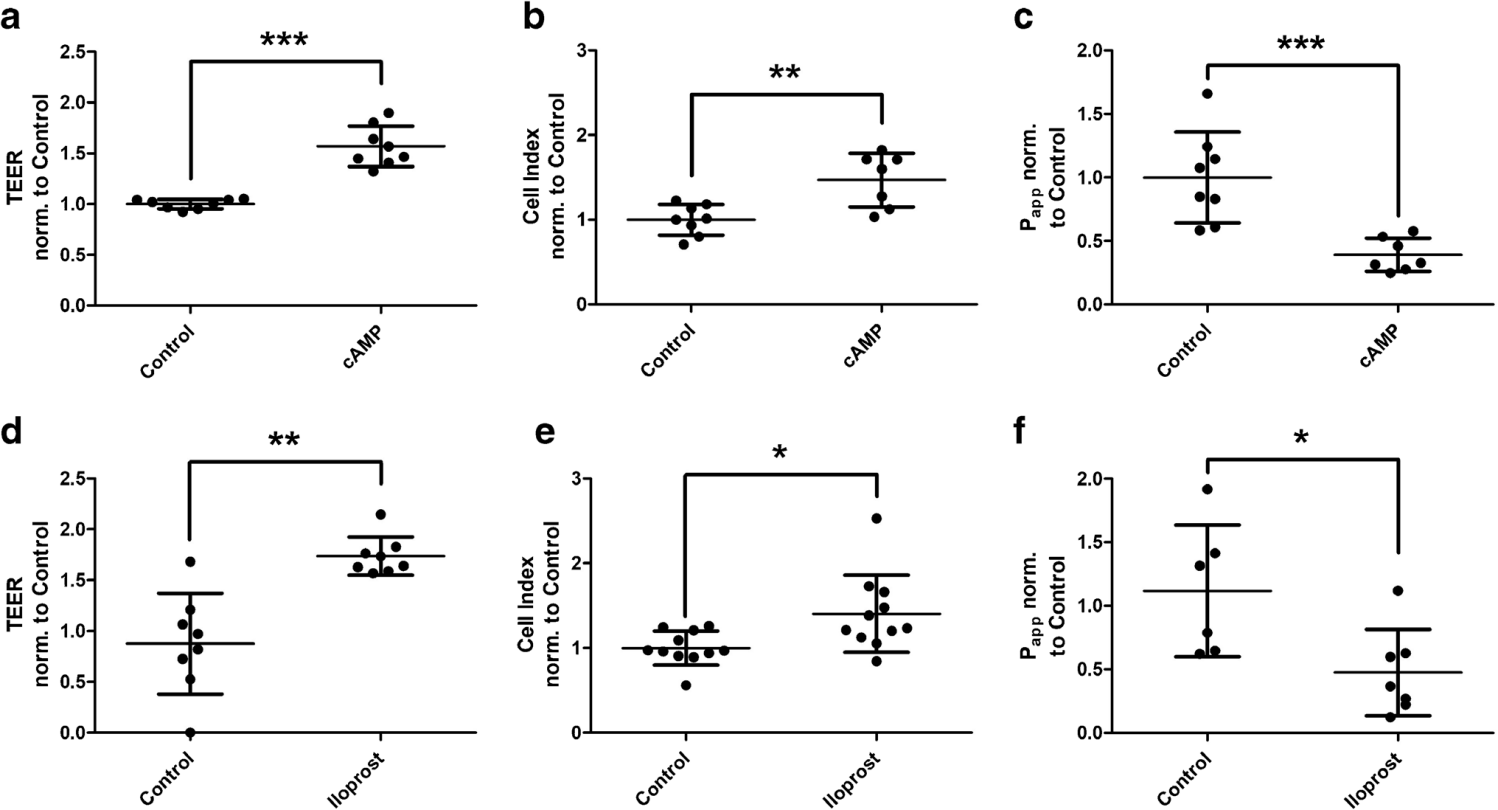
Measurements of endothelial barrier function under barrier-protective modulators. Human umbilical vein endothelial cells were cultured on semipermeable transwell filter systems and the modulators 8-bromo-adenosine-3′,5′-cyclophosphate (cAMP, **a-c**) and iloprost (**d-f**) were added on day three. After four hours, transendothelial electrical resistance (TEER, **a**/**d**), electrical capacity by determination of cell index (**b**/**e**), and apparent permeability coefficient (P_app_, **c**/**f**) were measured. The addition of the modulators resulted in an increased TEER, as well as an increased capacity, furthermore the P_app_ decreased significantly. Values are shown as scattered plots, and the mean value is additionally indicated with the standard deviation. Each data point represents one filter. Significances were calculated using the two-tailed Mann Whitney test (* = *p* < 0.05, ** = *p* < 0.01, *** = *p* < 0.001)

To confirm the effect of 8Br-cAMP and iloprost, TEER (Fig. 6a/d), capacity (Fig. 6b/e, expressed as cell index), and P_app_ (Fig. 6c/f) were additionally determined. All demonstrated the barrier-promoting effect of the compounds and thus supported the measurements of the D_2_O dilution method.

### Thrombin, histamine and bradykinin lead to increased transendothelial water movement

**Fig. 7:**
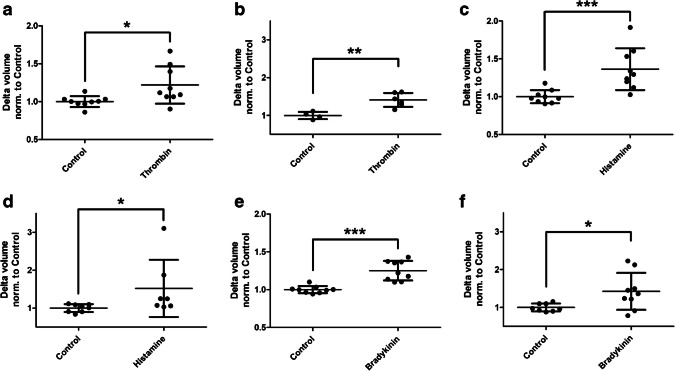
The D_2_O dilution method detects increased water movement under barrier-lowering modulators. Human umbilical vein endothelial cells were cultured on semipermeable transwell filter systems and exposed to a hydrostatic pressure of 1.0 cmH_2_O apically (**a**/**c**/**e**) and 0.75 cmH_2_O basally (**b**/**d**/**f**) on day three. The modulators thrombin (**a**/**b**), histamine (**c**/**d**), and bradykinin (**e**/**f**) were added four hours before measurement. Volume of the transendothelial moved fluid was determined by D_2_O dilution method. For all conditions a significantly increased water movement could be detected by addition of the modulators. The values are shown as scattered plots, and the mean value with the standard deviation is also given. Each data point represents one filter. Significances were calculated using the two-tailed Mann Whitney test (* = *p* < 0.05, ** = *p* < 0.01, *** = *p* < 0.001)

The enzyme thrombin, as well as the two tissue hormones histamine and bradykinin, are known to increase endothelial permeability. They were therefore used to further verify the D_2_O dilution method. Again, a basal (Fig. 7a/c/e) and apical (Fig. 7b/d/f) hydrostatic gradient were applied. Thrombin (Fig. 7a/b) led to a significantly increased water movement in each of the hydrostatic gradients. Histamine (Fig. 7c/d) and bradykinin (Fig. 7e/f) significantly increased water movement in both gradients.

### The barrier-lowering effect of thrombin, histamine and bradykinin can be verified by other methods

**Fig. 8:**
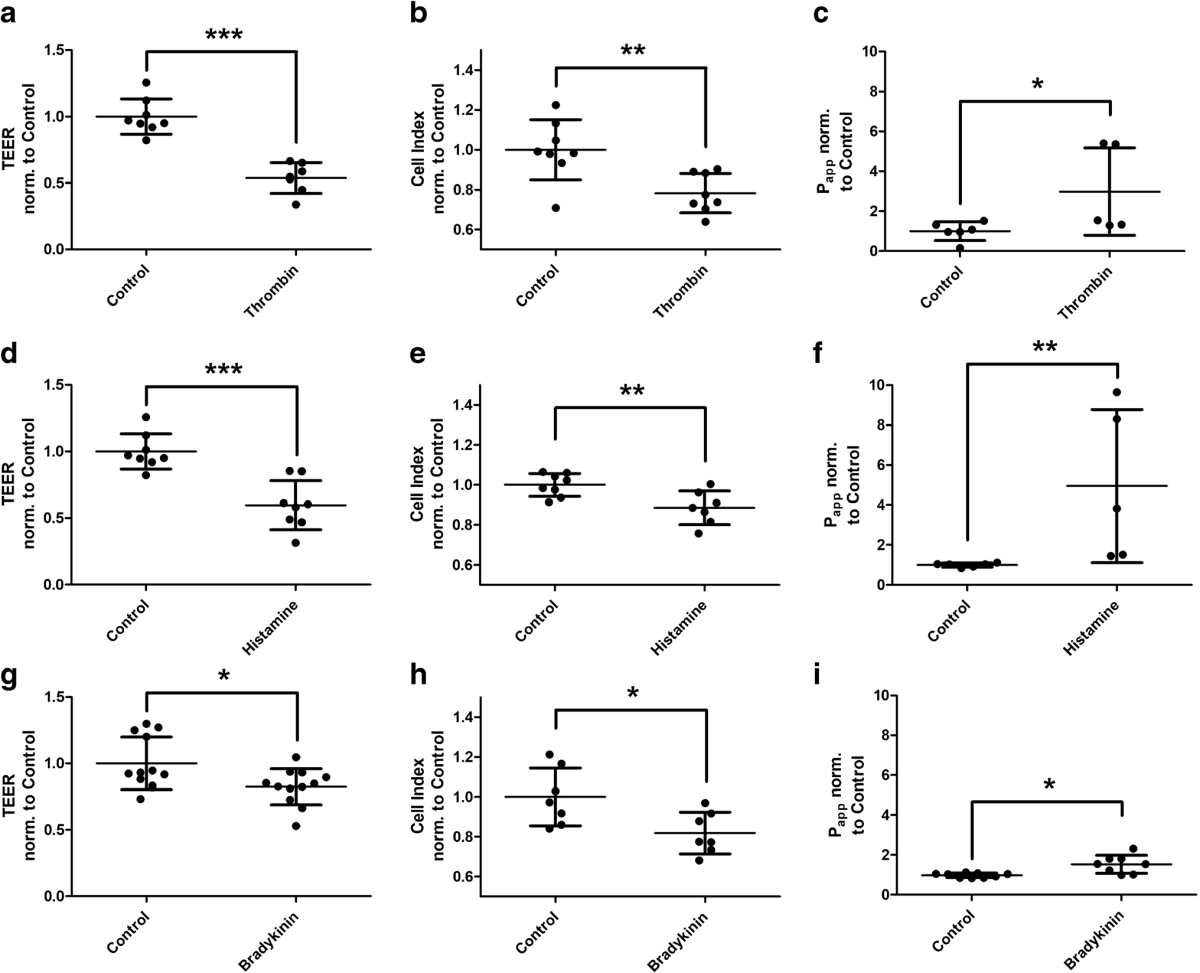
Measurements of endothelial barrier function under barrier-lowering modulators. Human umbilical vein endothelial cells were cultured on semipermeable transwell filter systems and the modulators thrombin (**a-c**), histamine (**d-f**) and bradykinin (**g-i**) were added on day three. After four hours, transendothelial electrical resistance (TEER, **a**/**d**/**g**), electrical capacity by determination of cell index (**b**/**e**/**h**), and apparent permeability coefficient (P_app_, **c**/**f**/**i**) were measured. The addition of the modulators resulted in a decreased TEER, as well as a decrease in capacity, and P_app_ also increased significantly. Values are shown as scattered plots, and the mean is also indicated with the standard deviation. Each data point represents one filter. Significances were calculated using the two-tailed Mann Whitney test (* = *p* < 0.05, ** = *p* < 0.01, *** = *p* < 0.001)

To confirm the results of the D_2_O dilution method, measurements of TEER (Fig. 8a/d/g), electrical capacity (Fig. 8b/e/h, given as cell index) and P_app_ were also performed for the three substances. These all confirmed the permeability increasing effect of thrombin (Fig. 8a/b/c), histamine (Fig. 8d/e/f) and bradykinin (Fig. 8g/h/i) and thus the D_2_O dilution method.

## Discussion

In blood vessels, the flow of water between the intravascular space and the interstitium is significantly modulated by the endothelium [8, 15]. Edema arises from a discrepancy between intra- and extravascular pressures, coupled with an augmented permeability of the endothelium [23]. Endothelial hyperpermeability also exacerbates various diseases, such as solid tumors [30] or acute respiratory failure [32]. The endothelial barrier function itself can be relatively accurately determined *in vitro*. Available methods include the measurement of TEER through frequency-dependent impedance differences [5], the assessment of electrical capacity using Real-Time Cell Analysis [17], and the determination of the apparent permeability coefficient for various marker substances [38]. However, the assessment of transendothelial water movement has been inadequately addressed *in vitro*. Existing methods, including gravimetry, microscopy, and absorption spectroscopy, suffer from limitations in accuracy and reliability [32, 36]. Gravimetry relies on the weight of the sample to deduce the underlying volume [16], but it fails when more precise volume determinations are required. Residual volumes on pipette tips or well walls introduce relevant sources of error, leading to inaccuracies [33]. Additionally, liquid volume determination using marker substances, as employed in absorption spectroscopy [22], has its drawbacks. Marker substances may undergo metabolism by cells [39], or exhibit altered absorption behavior based on pH [28]. These limitations underscore the necessity for a more accurate and less error-prone method to precisely determine transendothelial water movement. An advantageous method in this context is the D_2_O dilution method. Originally designed to determine volume shifts of lung epithelial cells with precision, this method capitalizes on differences in intramolecular vibrations between H_2_O and D_2_O. By introducing a known volume of D_2_O and measuring the exact ratio of H_2_O to D_2_O using infrared spectroscopy, one can ascertain the precise amount of H_2_O present [33]. In this study, the D_2_O dilution method will be employed to determine transendothelial water movements with the utmost accuracy.

Initially, the experimental setup was established, employing Human Umbilical Vein Endothelial Cells (HUVEC) as a widely accepted model for endothelial cells [20]. These cells were exposed to varying hydrostatic pressures from both the apical and basal directions, and the resultant transendothelial water movement was quantified. To rule out the possibility of a significant water flow even without hydrostatic gradients, control measurements were carried out without pressure. These showed no relevant volume shift after 24 h. The hydrostatic pressure gradients exhibited a linear correlation with water movement in both directions. Subsequently, to generate the highest possible water movement, an incubation time of 24 h and a hydrostatic pressure of 1 cmH_2_O apically and 0.75 cmH_2_O basally were selected for the following experiments. It is noteworthy that the hydrostatic pressures in the human body are considerably higher. The average hydrostatic pressure in the capillaries of the systemic circulation is approximately 25 mmHg, equivalent to roughly 34 cmH_2_O. *In vitro*, however, an artificial system is employed, focusing solely on measuring the water permeability of the endothelium, devoid of muscle cells, adventitia, or surrounding connective tissue. Consequently, water movement can be detected at significantly lower pressures, such as 0.5 cm H_2_O. The inverse pressure gradient from basal to apical of 0.75 cmH_2_O corresponds to about 0.5 mmHg, closely resembling the physiologically present pressure gradient from the interstitium to the vessel lumen. Time-course experiments revealed that the majority of fluid traverses the monolayer within the first hour, reaching a plateau at around two hours. This indicates that the D2O dilution method is capable of detecting volume shifts over a very short time period [21]. Notably TEER remained unchanged in both experimental setups despite the application of pressure gradients. Immunocytochemical experiments demonstrated intact cell-cell contacts following the application of hydrostatic pressure. Therefore, VE-CAD and ZO-1 were utilized as typical markers for adherens and tight junctions, respectively [37]. Consequently, it can be inferred that the experimental setup does not induce damage to the endothelial barrier.

For further verification of the D_2_O dilution method, additional endothelial cell types were analyzed. Besides venous HUVEC [4], arterial HUAEC [4] and lymphatic HDMEC [14] were used. In addition, BOEC isolated from peripheral venous blood samples from healthy volunteers were examined. By comparing endothelial cells of different origin, it is possible to shed light on further aspects and analyze differences between cells [1, 12, 18]. In all four cell types, water movements were observed at the applied pressure gradients, with HUVEC demonstrating higher water permeability compared to the other cell types. Furthermore, gravimetry and absorption spectroscopy were employed to reassess the functionality of the experimental setup. Gravimetry indicated a correspondingly directed water movement for both pressure gradients. However, the hydrostatic pressure gradient from apical to basal exhibited significantly lower movement than that measured with the D_2_O dilution method. This observation may be attributed to unweighed residual fluid volumes, as previously mentioned [33]. Absorption spectroscopy also broadly confirmed the experimental setup. However, this showed significantly higher delta volume values for an apically applied hydrostatic pressure than the other two methods. Furthermore, no water movement could be detected in the basally applied hydrostatic pressure. The possible metabolization or interaction of the phenol red used as marker substance, as well as its pH sensitivity, can be addressed as possible causes [26, 28, 39]. Overall, these methods confirmed the functionality of the experimental setup, albeit without achieving the precision and resolution of the D_2_O dilution method. Finally, we investigated whether modulations of the endothelial barrier lead to altered water movements and whether such changes can be detected by the D_2_O dilution method. To assess this, pharmacological modulators of the barrier were added to HUVEC. Initially, barrier-enhancing substances were used. Barrier increase occurs primarily via an intracellular increase in cAMP. This activates further signaling cascades that ultimately promote the formation of cortical actin bundles. This in turn reduces mechanical traction forces on cell-cell contacts [2]. In this work, the membrane-permeable cAMP analogue 8-Br-cAMP [9] and the prostacyclin analogue iloprost [13] were used, the latter causing an intracellular cAMP increase [34]. The efficacy of both compounds was verified by measuring significantly increased TEER and electrical capacity, as well as significantly decreased P_app_. Using the D_2_O dilution method, it could be shown that this increase in permeability also resulted in significantly decreased transendothelial water movement. This was demonstrated for both substances and both pressure gradients. Subsequently, the effect of permeability increasing modulators was investigated. Here, the enzyme thrombin, as well as the two peptide hormones histamine and bradykinin were used. All three bind to Gq/G11 protein-coupled receptors and thereby activate intracellular signaling cascades. This leads to increased actin-myosin contraction and phosphorylation of various components of adherens junctions [6, 40]. Again, their efficacy was verified by measuring TEER, capacity and P_app_. TEER and capacity decreased significantly for all three modulators, whereas P_app_ rose, indicating an overall increase in endothelial permeability. In turn, the D_2_O dilution method demonstrated that all modulators resulted in increased transendothelial water movement at the corresponding gradients. These results agree with other studies showing increased permeability by thrombin [35], histamine [3] and bradykinin [23]. Bradykinin exerts its function mainly through the bradykinin B_2_ receptor (B2R) [24]. The functionality of the bradykinin B2 receptor (B2R) was confirmed in our endothelial cell model by the addition of the specific B2R antagonist icatibant [24], which completely inhibited the TEER drop induced by bradykinin (Online Resource).

In conclusion, it is imperative to address the limitations of the current methodology in greater detail. Firstly, most of the current measurements were taken at one point in time (24 h), primarily due to the early stages of method establishment. Time course measurements unequivocally illustrated that a significant proportion of transendothelial water flow occured within the initial hour, reaching a plateau by the 24th hour. Many modulators of the endothelial barrier manifest their effects within minutes rather than hours [41]. Nonetheless, effects on transendothelial water flow were detectable, as corroborated by other studies on endothelial barrier function. To provide more precise insights, subsequent experiments should encompass additional time intervals. Currently, the method reflects complex cellular responses to modulators, presumably following the initial endothelial reaction. However, the time course underscores the suitability of the D_2_O dilution method for mapping such short time intervals, rendering it a valuable tool for future investigations into these questions. The experiments carried out here were also only performed on a limited number of biological replicates. More experiments with a larger number of samples are therefore necessary for final verification. The used endothelia show a comparatively high permeability. This is certainly due to the longer incubation time mentioned above, but also to the artificial system, which currently only consists of an endothelial monolayer cultivated on plastic filters. Nevertheless, the D_2_O dilution method is an important supplementary method. Mechanistic studies in particular are usually carried out in cell culture and are then transferred to *in vivo* models. Regarding transendothelial water permeability, such investigations *in vitro* have not yet been sufficiently performed. In the future, the focus should therefore be on optimizing this system, for example using co-culture models. Furthermore, primarily hydrostatically induced water flows are currently being investigated using the D_2_O dilution method. In a second step, it is therefore advisable to establish the D_2_O dilution method for osmotically induced water flows using plasma proteins such as albumin.

Despite the aforementioned limitations, the precision of the D_2_O dilution method surpasses that of established methods. It remains unaffected by the confounding factors associated with gravimetry and microscopy, eliminating the need for marker substances and minimizing the risk of susceptibility to interference. The presented results unequivocally demonstrate the method's capability to accurately measure changes in transendothelial water movement.

## Conclusion

This newly established *in vitro* method for directly measuring transendothelial water permeability has numerous areas of application. In particular, its accuracy and insusceptibility to interfering factors is unmatched to date. It offers an important addition, for example, to basic edema research and can also be used to develop barrier-protective pharmaceuticals. The use of patient-specific endothelial cells like BOEC also enables the adaptation of this type of research to a specific patient population, which means that a further step in the direction of personalized medicine can be taken.

### Supplementary information

Below is the link to the electronic supplementary material.

Supplementary file1 (PDF 150 KB)

## Data Availability

Supplementary information can be found online.
