# Supplementary material for: Establishment of the deuterium oxide dilution method as a new possibility for determining the transendothelial water permeability
Source: Pflugers Arch. 2024 Mar 5;476(6):993–1005. doi: 10.1007/s00424-024-02934-z (PMC11139723; doi:10.1007/s00424-024-02934-z)
Supplement: Supplementary file 1 — Supplementary file1 (PDF 150 KB) [file 424_2024_2934_MOESM1_ESM.pdf]

Establishment of the Deuterium Oxide Dilution Method as a New Possibility for Determining the Transendothelial Water Permeability

Pflügers Archiv – European Journal of Physiology

Authors: Hannes Müller[1], Janina Hahn[1], Angelina Gierke[1], Robert Stark[1], Cornelia Brunner[1], Thomas K. Hoffmann[1], Jens Greve[1], Oliver Wittekindt[2], Robin Lochbaum[c1]

E-Mail: robin.lochbaum@uniklinik-ulm.de

[C] Corresponding Author

[1] Department of Otorhinolaryngology, Head and Neck Surgery, Ulm University Medical Center, Germany

[2] Department of General Physiology, Ulm University, Germany

Supplementary information

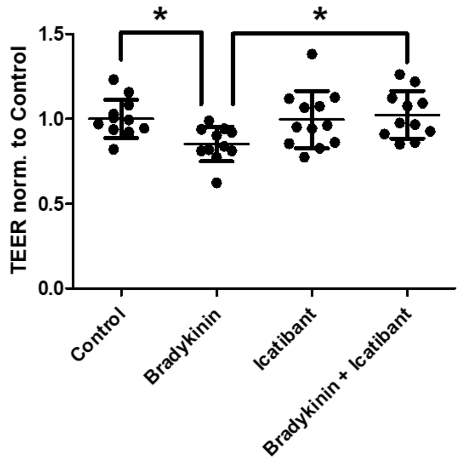

Supplemental figure: *Measurement of endothelial barrier function under bradykinin B_2 receptor modulation.* Human umbilical vein endothelial cells were cultured on semipermeable transwell filter systems and bradykinin as agonist, as well as icatibant as antagonist of bradykinin B_2 receptor were added. After four hours, transendothelial electrical resistance (TEER) was measured. Bradykinin reduced TEER, while icatibant alone had no impact on TEER. Combination of both significantly lowered the effect of bradykinin on TEER. Values are shown as scattered plots, and the mean is also indicated with the standard deviation. Each data point represents one filter, experiments were performed with two different cell batches. Significances were calculated using the two-tailed Mann Whitney test (* = $p < 0.05$, ** = $p < 0.01$, *** = $p < 0.001$)
